# Supplementary material for: The Development of a Novel Nanobody Therapeutic for SARS-CoV-2
Source: bioRxiv. 2020 Nov 17:2020.11.17.386532. Preprint. [Version 1] doi: 10.1101/2020.11.17.386532 (PMC7685322; doi:10.1101/2020.11.17.386532)
Supplement: 1 [file NIHPP2020.11.17.386532-supplement-1.pdf]

bioRxiv preprint doi: https://doi.org/10.1101/2020.11.17.386532; this version posted November 17, 2020. The copyright holder for this preprint (which was not certified by peer review) is the author/funder, who has granted bioRxiv a license to display the preprint in perpetuity. It is made available under aCC-BY 4.0 International license.

Supplementary materials for

"The Development of a Novel Nanobody Therapeutic for SARS-CoV-2"

Gang Ye [1,*], Joseph P. Gallant [2,*], Christopher Massey [3], Ke Shi [4], Wanbo Tai [5], Jian Zheng [6], Abby E. Odle [6], Molly A. Vickers [6], Jian Shang [1], Yushun Wan [1], Aleksandra Drelich [7], Kempaiah R. Kempaiah [7], Vivian Tat [8], Stanley Perlman [6], Lanying Du [5], Chien-Te Tseng [7,9], Hideki Aihara [4], Aaron M. LeBeau [2,#], Fang Li [1,#]

bioRxiv preprint doi: https://doi.org/10.1101/2020.11.17.386532; this version posted November 17, 2020. The copyright holder for this preprint (which was not certified by peer review) is the author/funder, who has granted bioRxiv a license to display the preprint in perpetuity. It is made available under aCC-BY 4.0 International license.

Table S1. X-ray data collection and structure refinement statistics (SARS-CoV-2 RBD/*Nanosota-1C* complex)

**Data collection**	
Wavelength	0.979
Resolution range	45.48 - 3.19 (3.30 - 3.19)
Space group	P 43 21 2
Unit cell	60.849 60.849 410.701 90 90 90
Total reflections	64167 (5703)
Unique reflections	13607 (1308)
Multiplicity	4.7 (4.4)
Completeness (%)	96.82 (97.60)
Mean I/sigma(I)	8.41 (1.80)
Wilson B-factor	83.24
R-merge	0.145 (0.928)
R-meas	0.1638 (1.053)
R-pim	0.07385 (0.4858)
CC1/2	0.995 (0.861)
CC*	0.999 (0.962)
**Refinement**	
Reflections used in refinement	13567 (1301)
Reflections used for R-free	674 (62)
R-work	0.2483 (0.3521)
R-free	0.2959 (0.4153)
CC(work)	0.963 (0.819)
CC(free)	0.909 (0.615)
Number of non-hydrogen atoms	4890
macromolecules	4833
ligands	57
Protein residues	621
RMS(bonds)	0.002
RMS(angles)	0.45
Ramachandran favored (%)	93.11
Ramachandran allowed (%)	6.89
Ramachandran outliers (%)	0.00
Rotamer outliers (%)	3.23
Clashscore	5.25
Average B-factor	90.29
macromolecules	89.84
ligands	127.91

Statistics for the highest-resolution shell are shown in parentheses.

bioRxiv preprint doi: https://doi.org/10.1101/2020.11.17.386532; this version posted November 17, 2020. The copyright holder for this preprint (which was not certified by peer review) is the author/funder, who has granted bioRxiv a license to display the preprint in perpetuity. It is made available under aCC-BY 4.0 International license.

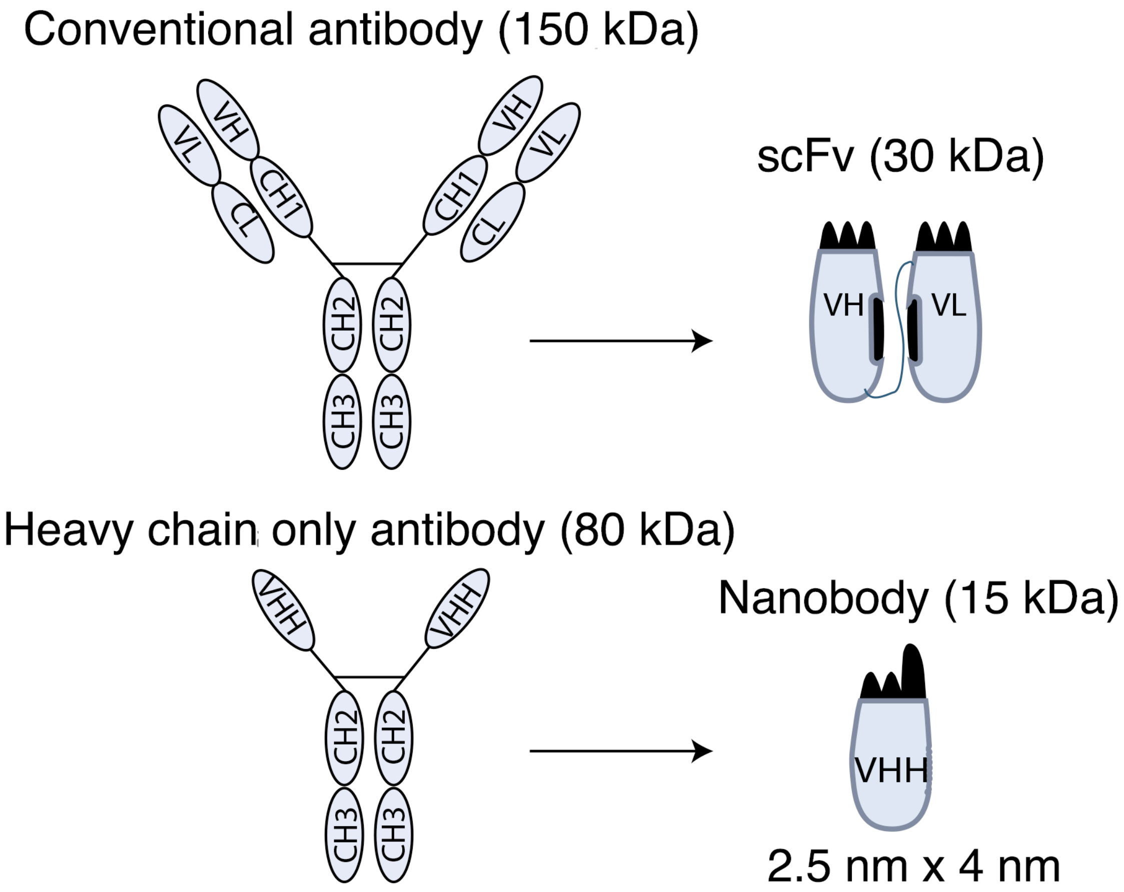

Figure S1. Schematic drawings of nanobodies and conventional antibodies. VH: variable domain of heavy chain. CH: constant domain of heavy chain. VL: variable domain of light chain. CL: constant domain of light chain. VHH: variable domain of heavy-chain only antibody. scFv: single-chain variable fragment.

bioRxiv preprint doi: https://doi.org/10.1101/2020.11.17.386532; this version posted November 17, 2020. The copyright holder for this preprint (which was not certified by peer review) is the author/funder, who has granted bioRxiv a license to display the preprint in perpetuity. It is made available under aCC-BY 4.0 International license.

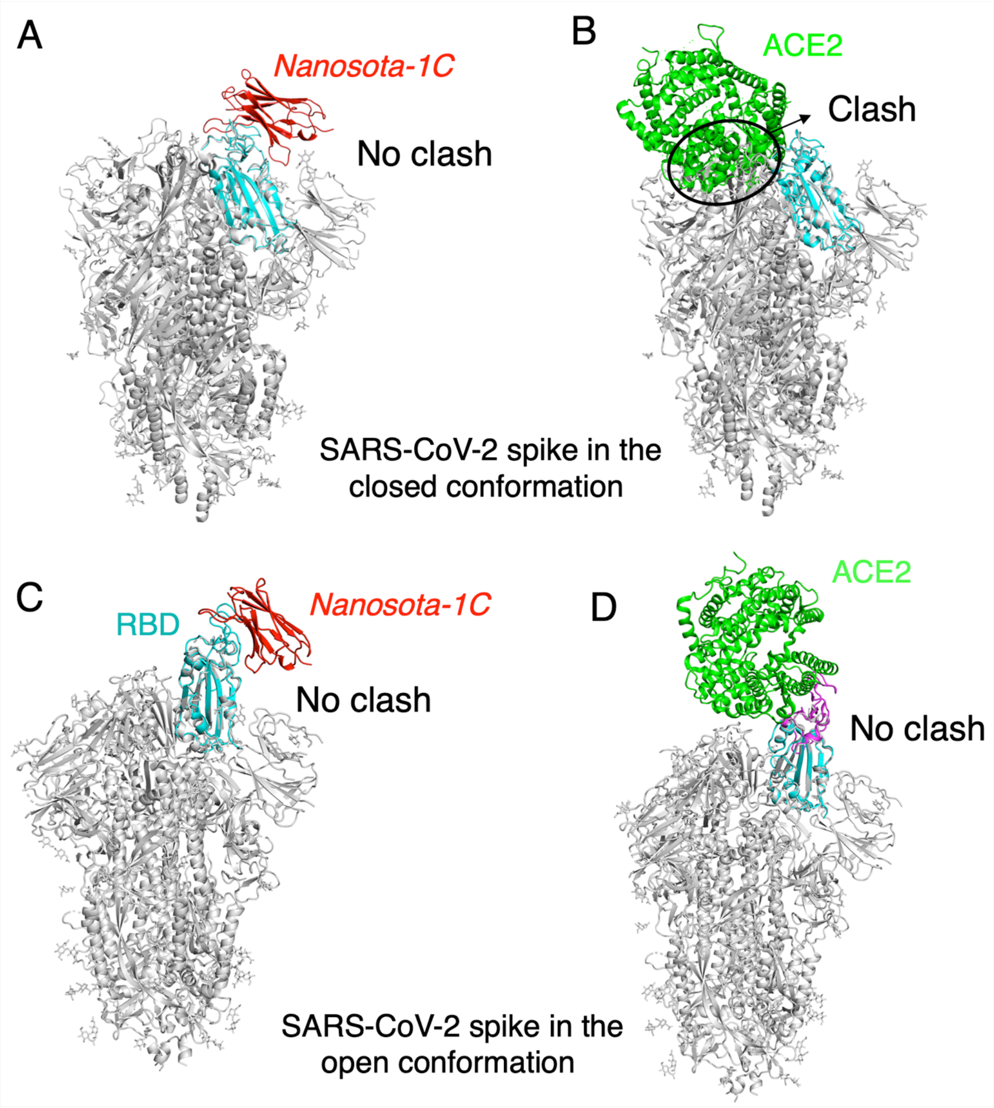

Figure S2. The binding of *Nanosota-1C* to SARS-CoV-2 spike protein in different conformations. (A) The binding of *Nanosota-1C* to the spike protein in the closed conformation. The structures of the RBD/*Nanosota-1C* complex and SARS-CoV-2 spike protein in the closed conformation (PDB: 6ZWV) were superimposed based on their common RBD structure (in cyan). *Nanosoto-1C* is in red. The rest of the spike protein is in gray. (B) The binding of ACE2 to the spike protein in the closed conformation. The structures of the RBD/ACE2 complex (PDB 6M0J) and SARS-CoV-2 spike protein in the closed conformation (PDB: 6ZWV) were superimposed based on their common RBD structure. ACE2 is in green. Clashes between ACE2 and the rest of the spike protein were circled. (C) The binding of *Nanosota-1C* to the spike protein in the open conformation (PDB: 6VSB). (D) The binding of ACE2 to the spike protein in the open conformation (PDB: 6VSB).

bioRxiv preprint doi: https://doi.org/10.1101/2020.11.17.386532; this version posted November 17, 2020. The copyright holder for this preprint (which was not certified by peer review) is the author/funder, who has granted bioRxiv a license to display the preprint in perpetuity. It is made available under aCC-BY 4.0 International license.

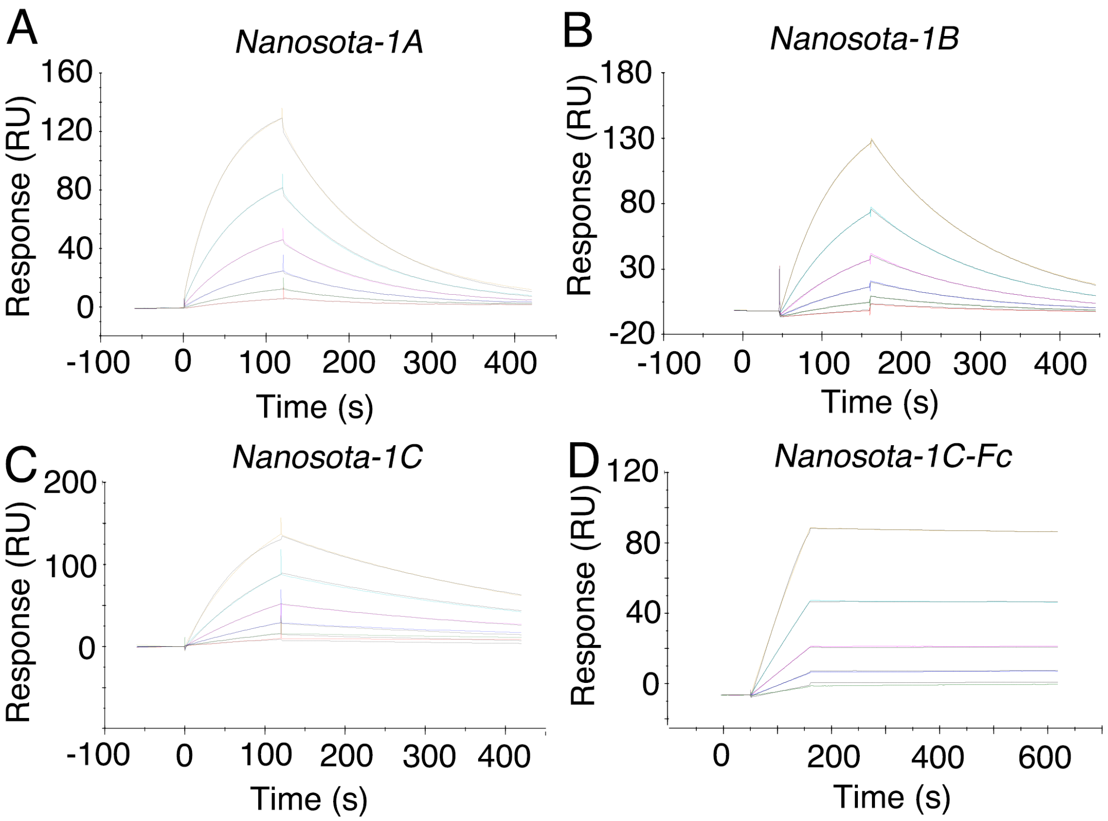

Figure S3. Measurement of the binding affinities between *Nanosota-1* drugs and SARS-CoV-2 RBD by surface plasmon resonance assay using Biacore. Purified recombinant SARS-CoV-2 RBD was covalently immobilized on a sensor chip through its amine groups. Purified recombinant nanobodies flowed over the RBD individually at one of five different concentrations. The resulting data were fit to a 1:1 binding model and the value of K_d was calculated for each nanobody. The assay was repeated three times (biological replication: new aliquots of proteins and new sensor chips were used for each repeat).

bioRxiv preprint doi: https://doi.org/10.1101/2020.11.17.386532; this version posted November 17, 2020. The copyright holder for this preprint (which was not certified by peer review) is the author/funder, who has granted bioRxiv a license to display the preprint in perpetuity. It is made available under aCC-BY 4.0 International license.

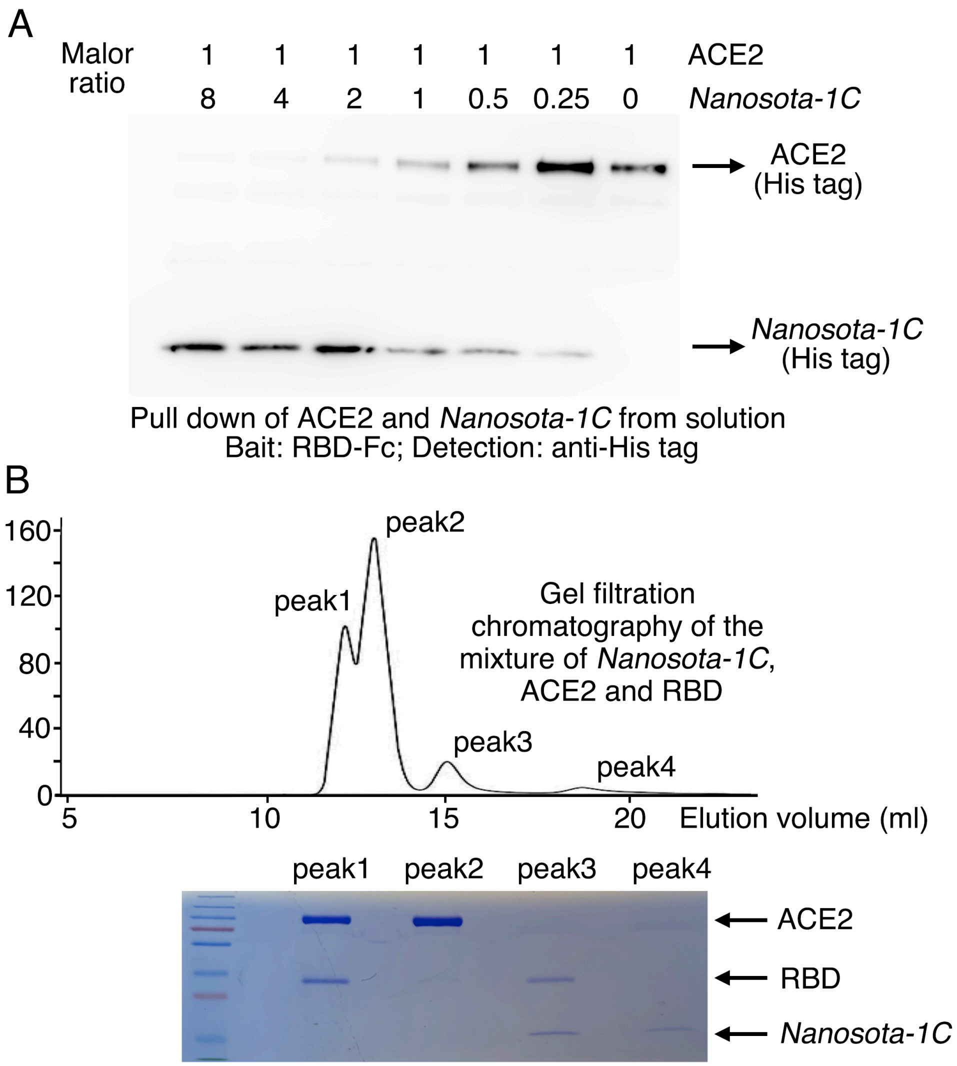

Figure S4. Binding interactions between *Nanosota-1* drugs and SARS-CoV-2 RBD. (A) Binding interactions between SARS-CoV-2 RBD, *Nanosota-1C*, and ACE2 as evaluated using a protein pull-down assay. Various concentrations of *Nanosota-1C* and a constant concentration of ACE2 (all His tagged) were combined in different molar ratios. SARS-CoV-2 RBD (Fc tagged) was used to pull down *Nanosota-1C* and ACE2. A western blot was used to detect the presence of *Nanosota-1C* and ACE2 following pull down by SARS-CoV-2 RBD. The assay was repeated three times (biological replication: new aliquots of proteins were used for each repeat). (B) Binding interactions between SARS-CoV-2 RBD, *Nanosota-1C,* and ACE2 as examined using gel filtration chromatography. *Nanosota-1C*, ACE2 and SARS-CoV-2 RBD (all His tagged) were mixed together in solution (both *Nanosota-1C* and ACE2 in molar excess of SARS-CoV-2 RBD) and purified using gel filtration chromatography. Protein components in each of the gel filtration chromatography peaks were analyzed with SDS-PAGE and stained by Coomassie blue. The assay was repeated three times (biological replication: new aliquots of proteins were used for each repeat).

bioRxiv preprint doi: https://doi.org/10.1101/2020.11.17.386532; this version posted November 17, 2020. The copyright holder for this preprint (which was not certified by peer review) is the author/funder, who has granted bioRxiv a license to display the preprint in perpetuity. It is made available under aCC-BY 4.0 International license.

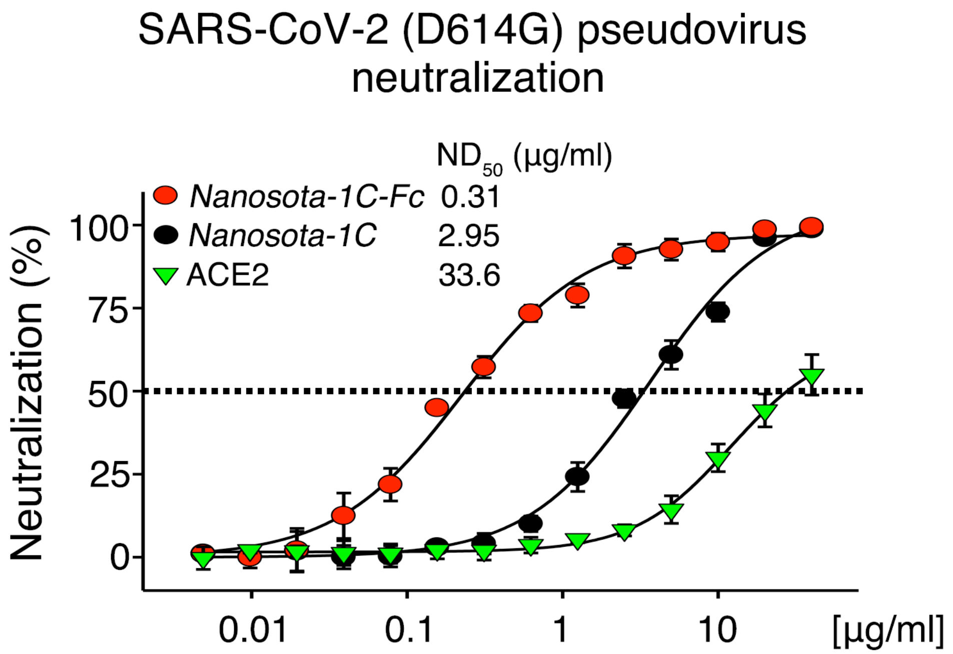

Figure S5. Neutralization of SARS-CoV-2 pseudovirus, which contains the D614G mutation in the spike protein, by *Nanosota-1* drugs. The procedure was the same as described in Fig. 3A, except that the mutant spike protein replaced the wild type spike protein. The assay was repeated three times (biological replication: new aliquots of pseudoviruses and cells were used for each repeat).

bioRxiv preprint doi: https://doi.org/10.1101/2020.11.17.386532; this version posted November 17, 2020. The copyright holder for this preprint (which was not certified by peer review) is the author/funder, who has granted bioRxiv a license to display the preprint in perpetuity. It is made available under aCC-BY 4.0 International license.

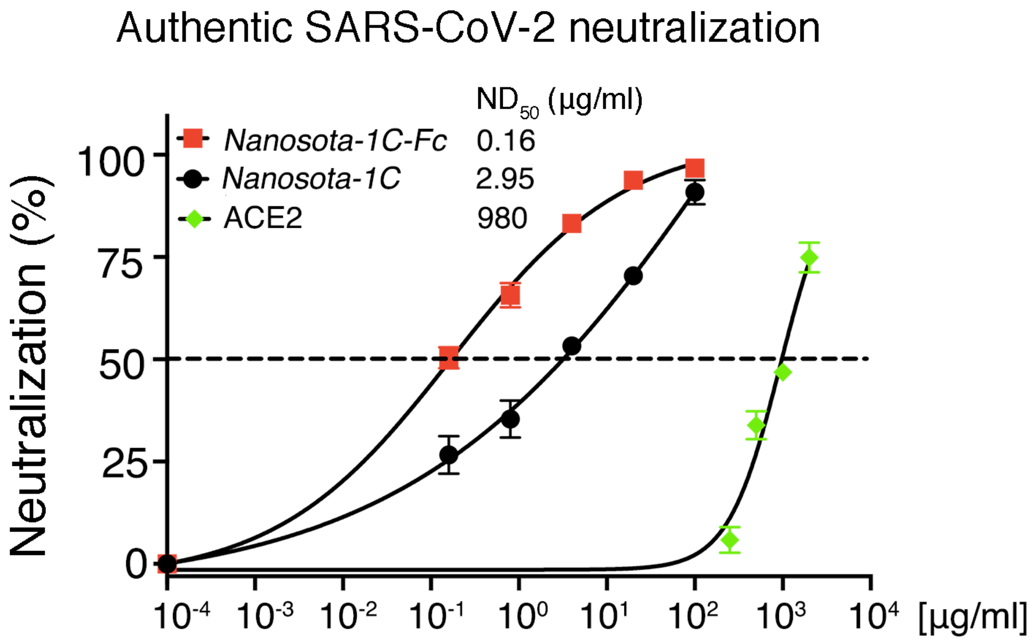

Figure S6. Detailed data on the neutralization of authentic SARS-CoV-2 infection of target cells by *Nanosota-1* drugs. Data are the mean ± SEM (n = 3). Nonlinear regression was performed using a log (inhibitor) versus normalized response curve and a variable slope model ($R^2 > 0.95$ for all curves). The assay was repeated twice (biological replication: new aliquots of virus particles and cells were used for each repeat).

bioRxiv preprint doi: https://doi.org/10.1101/2020.11.17.386532; this version posted November 17, 2020. The copyright holder for this preprint (which was not certified by peer review) is the author/funder, who has granted bioRxiv a license to display the preprint in perpetuity. It is made available under aCC-BY 4.0 International license.

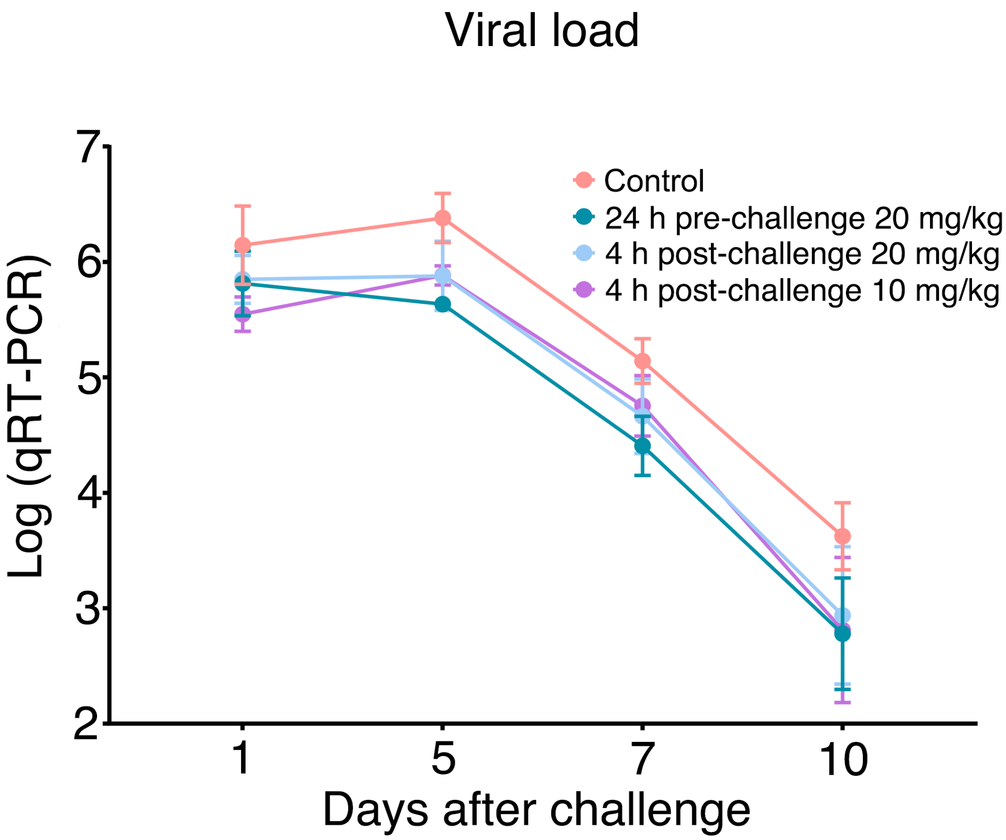

Figure S7. Additional data on the efficacy of *Nanosota-1* drugs in protecting hamsters from SARS-CoV-2 infections. Nasal swabs were collected from each hamster on days 1, 2, 3, 5, 7, and 10. Nasal swab samples from day 2 and day 3 were lost due to Hurricane Laura. qRT-PCR was performed to determine the virus loads in each of the samples. The qRT-PCR results are displayed on a log scale (since qRT-PCR amplifies signals on a log scale). Data are the mean ± SEM (n = 6). Missing data from one animal in the 4-hour post-challenge (10mg/kg) group on Day 7 were replaced by the average of that animal's days 5 and 10 data. ANOVA analysis using group as a between-group factor and day (1, 5, 7, and 10) as a within-group factor revealed significant differences between the control group and each of the following groups: 24 hour pre-challenge (20 mg/kg) group ($F(1, 10) = 6.02$, $p = .017$, effect size $\eta_p^2 = .38$), 4 hour post-challenge (20 mg/kg) group ($F(1, 10) = 5.38$, $p = .037$, $\eta_p^2 = .31$), and 4 hour post-challenge (10 mg/kg) group ($F(1, 10) = 3.40$, $p = .048$, $\eta_p^2 = .25$). All *p*-values are one-tailed for directional tests.

bioRxiv preprint doi: https://doi.org/10.1101/2020.11.17.386532; this version posted November 17, 2020. The copyright holder for this preprint (which was not certified by peer review) is the author/funder, who has granted bioRxiv a license to display the preprint in perpetuity. It is made available under aCC-BY 4.0 International license.

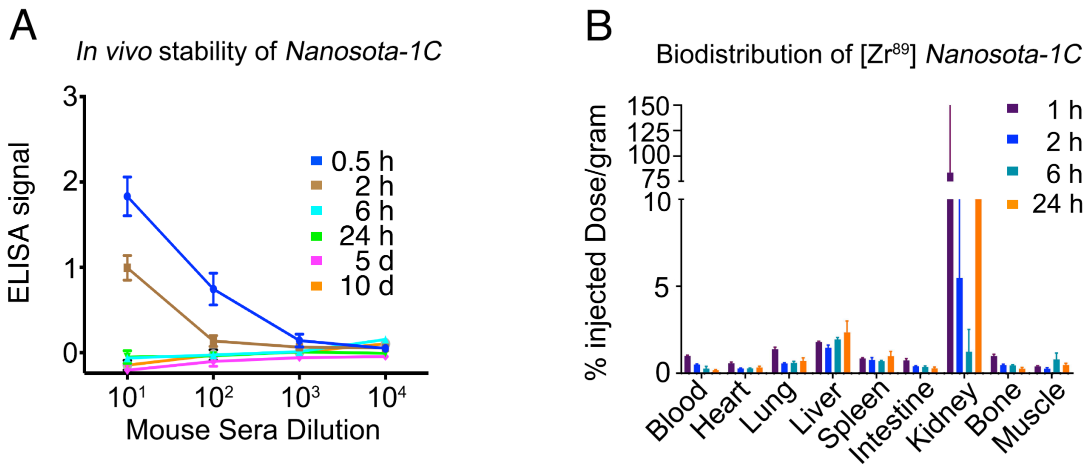

Figure S8. Pharmacokinetics of Nanosota-1C. *In vivo* stability and biodistribution of *Nanosota-1C* were measured in the same way as described in Fig. 5C and Fig. 5D, respectively, except that time points for *Nanosota-1C* differed from those for *Nanosota-1C-Fc* due to pharmacokinetic differences of the small molecular weight nanobody versus the larger Fc tagged nanobody.
